# Microglial SMAD4 regulated by microRNA-146a promotes migration of microglia which support tumor progression in a glioma environment

**DOI:** 10.18632/oncotarget.25116

**Published:** 2018-05-18

**Authors:** Aparna Karthikeyan, Neelima Gupta, Carol Tang, Karthik Mallilankaraman, Maskomani Silambarasan, Meng Shi, Lei Lu, Beng Ti Ang, Eng-Ang Ling, S. Thameem Dheen

**Affiliations:** ^1^ Department of Anatomy, Yong Loo Lin School of Medicine, National University of Singapore, Singapore; ^2^ Department of Physiology, Yong Loo Lin School of Medicine, National University of Singapore, Singapore; ^3^ School of Biological Sciences, Nanyang Technological University, Singapore; ^4^ Department of Research, National Neuroscience Institute, Singapore; ^5^ Duke-NUS Medical School, Singapore; ^6^ Division of Cellular and Molecular Research, National Cancer Centre, Singapore; ^7^ Department of Neurosurgery, National Neuroscience Institute, Singapore; ^8^ Singapore Institute for Clinical Sciences, A*STAR, Singapore

**Keywords:** microglia, glioma, TGFβ, SMAD4, microRNA-146a

## Abstract

Glioma tumors constitute a significant portion of microglial cells, which are known to support tumor progression. The present study demonstrates that transforming growth factor-β (TGFβ) signaling pathway in microglia in a glioma environment is involved in tumor progression and pathogenesis. It has been shown that the TGFβ level is elevated in higher grades of gliomas and its signaling pathway regulates tumor progression through phosphorylation of SMAD2 and SMAD3, which form a complex with SMAD4 to regulate target gene transcription. In an *in vitro* cell line-based model increased protein levels of pSMAD2/3, total SMAD2/3 and SMAD4 were observed in murine BV2 microglia cultured in glioma conditioned medium (GCM), indicative of the activated TGFβ signaling pathway in microglia associated with glioma environment. Immunofluorescence labeling further revealed the expression of SMAD4 in microglial and non-microglial cells of human glioblastomas tissue *in vivo*. Functional analysis through shRNA-mediated stable knockdown of SMAD4 in microglia revealed the downregulation of the expression of matrix metalloproteinase 9 (MMP9), which has been shown to be involved in tumor progression and cell migration. Further, knockdown of SMAD4 in microglia decreased the migration of microglial cells towards GCM, indicating that SMAD4 promotes microglial migration in glioma environment. In addition, SMAD4 has been shown to be post-transcriptionally regulated by microRNA-146a, which was downregulated in microglia treated with GCM. Overexpression of miR-146a resulted in decreased expression of SMAD4 together with tumor supportive gene MMP9 in microglia, and subsequently suppressed microglial migration towards GCM, possibly through regulation of SMAD4. On the other hand, the cell viability assay revealed decreased viability of glioma cells when they were treated with conditioned medium derived from SMAD4 knockdown microglia or miR-146a overexpressed microglia as compared to glioma cells treated with the medium from control microglial cells. Taken together, the present study suggests that microglial SMAD4 which is epigenetically regulated by miR-146a promotes microglial migration in gliomas and glioma cell viability.

## INTRODUCTION

Gliomas are malignant brain tumors with a wide range of clinical features. Gliomas arise from the glial cells of the brain, progressing through the benign stage (WHO Grade I) to highly malignant (WHO Grades II to IV) stages. Higher grades of glioma are heterogeneous in nature, consisting of neoplastic cells, glioma-like stem cells, extensive vasculature and immune cells [1–3]. Microglia, the resident immune cells of the brain, form a significant portion of the infiltrating immune cell population in a tumor, making up to one-third of the tumor mass in some higher grade tumors [4, 5]. As the first responders to any injury, insult or infection in the central nervous system (CNS), microglia become activated, secrete a variety of pro-inflammatory cytokines and exhibit phagocytic activity to clear tissue debris [6]. Subsequently, microglia facilitate repair and regeneration of the affected region in the CNS *via* release of growth factors and anti-inflammatory cytokines [7, 8]. Activated microglia exhibit neurotoxic and neuroprotective roles in neuropathology and based on their functions, microglia are categorized as the classical (pro-inflammatory) phenotype and the alternative (anti-inflammatory) phenotype [9, 10]. Microglial function in glioma tumors is an alternative form of activation wherein microglia secrete cytokines and chemokines that are gliomagenic and support the growth of the tumor [11, 12]. However, recent studies suggest that tumor-associated microglia express genes that are distinct from either activation state [13, 14], thus emphasizing the complex nature of tumor-associated microglia and its roles in a glioma microenvironment. This tumorigenic nature of microglia in glioma tumors may be attributed to molecular and epigenetic pathways that are altered by signaling molecules released from cancerous cells in the microenvironment.

Neoplastic cells within a tumor secrete a number of soluble cytokines, chemokines and growth factors that affect microglial motility, proliferation and phagocytosis [15, 16]. A key signaling molecule that is highly enriched in the glioma microenvironment is the Transforming Growth Factor-beta (TGFβ) which activates the TGFβ pathway that is mediated by SMAD2 and 3, substrates for the TGFβ family of receptors. Upon binding of the TGFβ ligand to its receptor, the SMAD2/3 complex is phosphorylated and coupled with the common mediator SMAD4, translocated to the nucleus where the complex regulates the transcription of TGFβ responsive genes [17]. TGFβ is a known inhibitor of cell cycle progression [18] and thus, functions as a tumor suppressor in the early stages of certain cancers. On the contrary, TGFβ signaling can be pro-tumorigenic by inducing genes that promote tumorigenic aspects of glioma progression such as angiogenesis [19], metastasis [20, 21] and epithelial-mesenchymal transition [22]. Hyperactive TGFβ signaling is associated with certain subtypes of glioblastoma tumors, such as the mesenchymal subset and contributes to aggressiveness of the tumor and poor prognosis in patients [23–25]. In tumors with activated TGFβ signaling such as hepatocellular cancer, elevated SMAD4 has been shown to mediate tumor promoting signaling [26], while in other cancers such as pancreatic cancer, deletion of SMAD4 is associated with tumor progression and metastasis [27, 28]. Therapeutic approaches using TGFβ antagonists and oligonucleotides coding anti-sense TGFβ2 have proven successful in reversal of TGFβ-aided immunosuppression in glioma [29, 30]. However, systemic inhibition of TGFβ pathway can lead to unfavorable effects as TGFβ is involved in several cellular signaling pathways. This led us to investigate alternate specific mechanisms by which the TGFβ signaling pathways can be disrupted to attenuate the tumor supportive phenotype of microglia. Moreover, the role of SMAD4 in microglial functions in gliomas has been poorly understood and hence, this study is aimed to understand the role of SMAD4 in tumor-associated microglia in mediating tumor progression.

In addition to altered signaling pathways, activated microglia in different neuropathologies exhibit dysregulated epigenetic mechanisms such as chromatin modifications, changes in gene-specific histone acetylation and methylation and differential microRNA (miRNA) expression [31, 32]. In particular, miRNAs have emerged as a central class of epigenetic mediators that post-transcriptionally regulate gene expression [33]. Dysregulation of miRNAs in activated microglia has been shown to contribute to development and progression of neurodegenerative diseases and brain injuries [33]. A global miRNA microarray analysis of activated primary microglial cells identified several miRNAs that were differentially expressed in activated microglia. The micro RNA 146a (miR-146a) was found to be upregulated in activated microglia as compared to control microglia (unpublished data). MiR-146a, which is enriched in activated macrophages and microglia [34], has been shown to target and suppress mediators of the nuclear factor kappa-light-chain-enhancer of activated B cells (NFκB) signaling pathway in activated microglia and astrocytes, thereby functioning as a negative feedback regulator of microglial activation [35, 36]. In addition, miR-146a was reported to target Notch1 in glioma cells and further inhibit the process of gliomagenesis by suppressing migration and proliferation of cancer cells [37]. Further, our bioinformatics analysis predicted miR-146a to target SMAD4. Given the important role of miR-146a in microglia activation and gliomagenesis and its putative effect on SMAD4, this study attempted to understand the role of miR-146a and its putative target SMAD4 in microglia functions in tumor progression in glioma environment.

It was hypothesized that altered molecular and epigenetic mechanisms regulate the tumor supportive behavior of glioma-associated microglia. In this study, SMAD4, a mediator of the TGFβ signaling pathway was upregulated in microglia exposed to glioma conditioned medium and was found to be robustly expressed in microglia associated with human glioblastoma tissues. Stable loss of SMAD4 in microglia decreased expression level of a tumor promoter, MMP9, which resulted in decreased migratory potential of microglia in a transwell migration assay. In addition, miR-146a which was predicted to target SMAD4, was downregulated in microglia exposed to glioma conditioned medium treatment and regulated the expression levels of tumor supportive gene MMP9 in microglia. Overall, the present study implicates the role of miR-146a-SMAD4 in regulating microglial functions in glioma tumors.

## RESULTS

### Glioma conditioned medium induced phosphorylation of SMAD2 and SMAD3 in microglia

**Figure 1:**
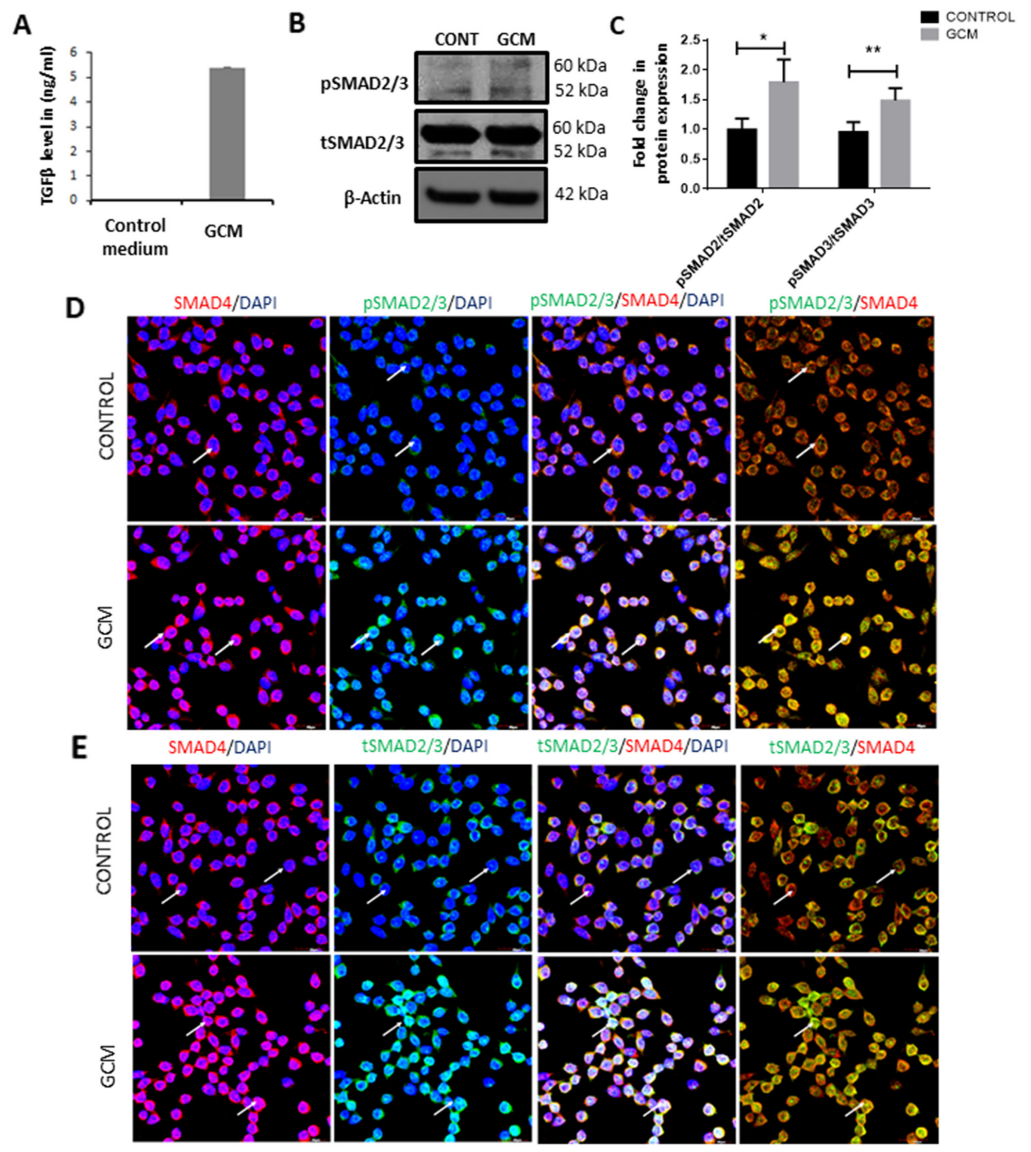
GCM induces TGFβ signaling pathway in microglia ELISA assay quantification revealed that GCM contains ~5ng/ml of TGFβ **(A)**. Western Blot **(B)** shows an increase in phosphorylated form of pSMAD2/3 in microglia treated with GCM when compared with control. Histogram depicts densitometric quantification of pSMAD2/3 normalized against total SMAD2/3 expression **(C)** Data represent mean±SD, (n=4), Students *t*-test, ^*^p<0.05, ^**^p<0.01. Confocal images show pSMAD2/3 (green, **D**), total SMAD2/3 expression (green, **E**) and SMAD4 expression (red, D, E) in BV2 microglia (*indicated by arrows*). Cell nuclei are labelled with DAPI (blue). GCM increases immunofluorescence intensity of pSMAD2/3 as compared to control cells (D). Immunocolocalization of pSMAD2/3, total SMAD2/3 and SMAD4 reveals that pSMAD2/3 and total SMAD2/3 colocalize with SMAD4 in microglia treated with GCM (D, E). Scale bar=30μm.

To understand the effect of glioma microenvironment on microglia, BV2 microglial cells were cultured in glioma conditioned medium (GCM) derived from C6 glioma cells. The concentration of TGFβ in the GCM was assessed to be ~5ng/ml as compared to undetectable levels of TGFβ in serum containing medium (Figure 1A). First, the expression of total and phosphorylated SMAD2/3, which mediates the TGFβ signaling, was determined in GCM-treated microglia. Western blot analysis showed an increase in the phosphorylated levels of SMAD2 and SMAD3 in GCM treated microglia as compared to control microglia (Figure 1B, 1C). Upon phosphorylation of SMAD2 and SMAD3, the complex has been shown to bind to SMAD4 and translocate to the nucleus [38]. Confocal imaging revealed a co-localization of the pSMAD2/3 with SMAD4 in nuclei of microglial cells treated with GCM (Figure 1D). Further, immunocytochemistry revealed increased co-localization of the SMAD4 and total SMAD2/3 in microglia treated with GCM as compared to control cells (Figure 1E), suggesting that the TGFβ pathway is activated in GCM-treated microglia.

### Microglia treated with GCM show increased expression of SMAD4 and tumor supportive genes

**Figure 2:**
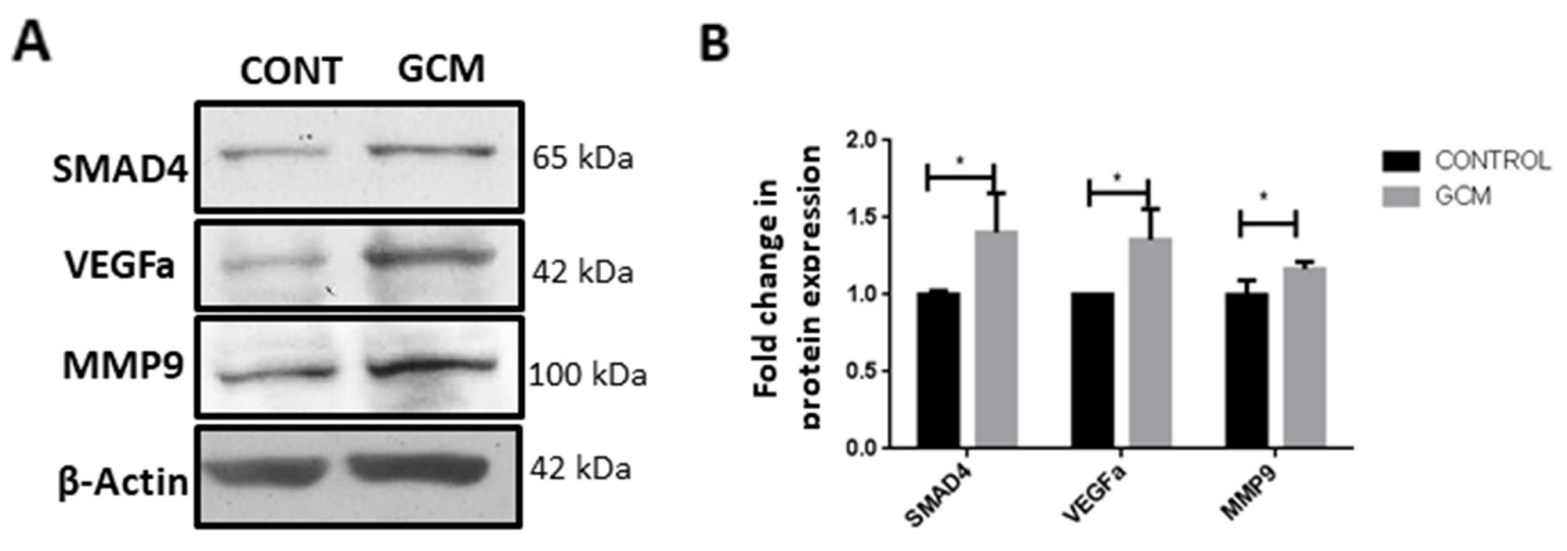
SMAD4 and tumor supportive genes MMP9 and VEGFa are upregulated in glioma associated microglia Western blot analysis shows an increase in SMAD4 protein levels and tumor supportive genes, VEGFa and MMP9 in BV2 microglia upon GCM treatment **(A)**. Histogram depicts densitometric quantification of protein levels of SMAD4, VEGFa and MMP9 in microglia exposed to GCM **(B)**. Data represent Mean±SD, (n=3-5), Students *t*-test, ^*^p<0.05.

As SMAD2/3 forms a complex with SMAD4 to regulate TGFβ responsive genes, the effect of glioma microenvironment on SMAD4 expression in microglia was determined by western blot and immunocytochemistry. Upregulation of SMAD4 protein expression was clearly evident in microglia treated with GCM as compared to that of control (Figure 2A, 2B). Concomitantly, GCM treated microglial cells showed an upregulation in proteins involved in TGFβ signaling pathway such as matrix metalloproteinase 9 (MMP9) and vascular endothelial growth factor (VEGFa) (Figure 2A, 2B), suggesting that glioma-associated microglia exhibit upregulated expression of tumor supportive factors.

### SMAD4 is expressed in microglia associated with human glioblastoma samples

**Figure 3:**
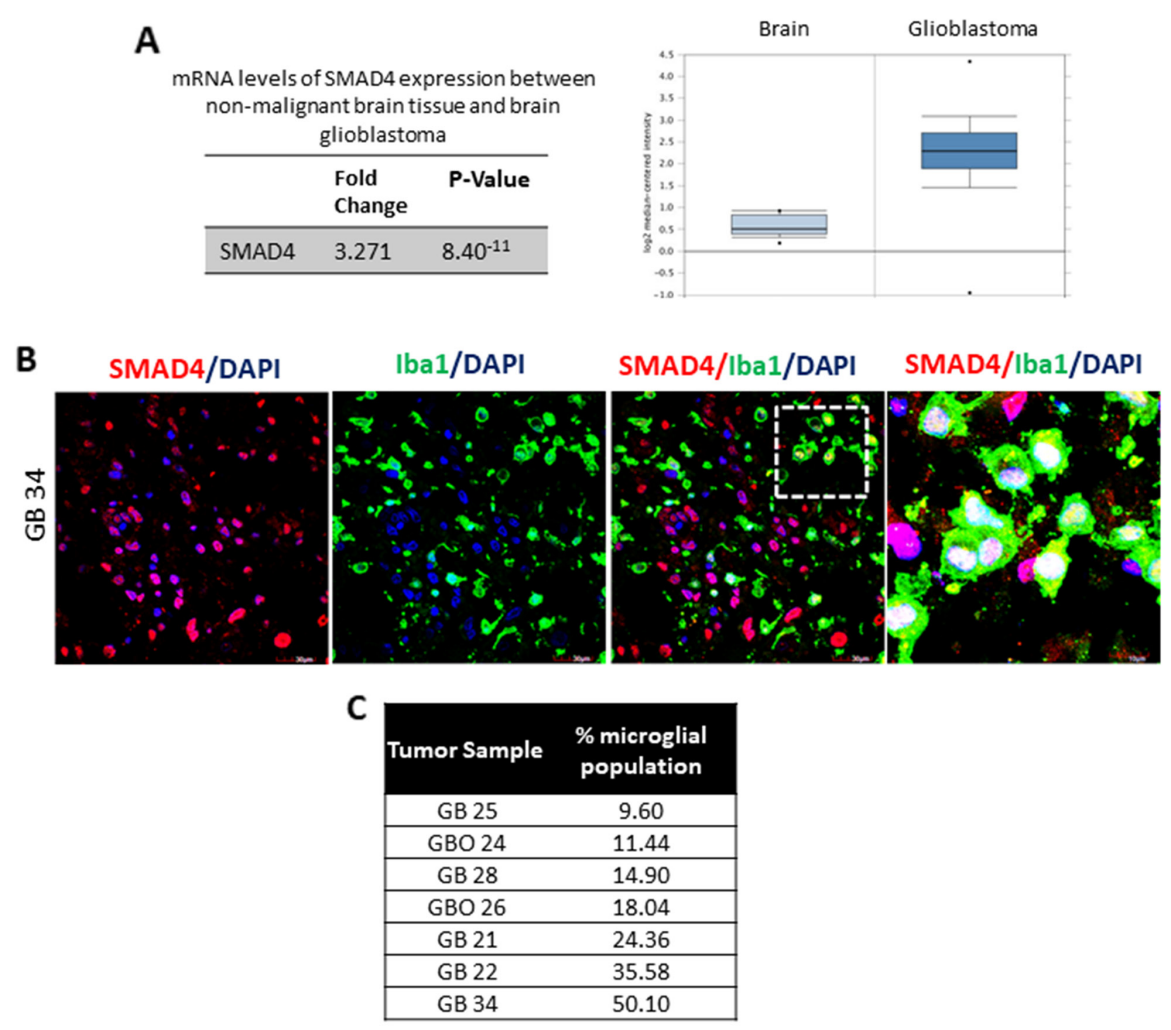
SMAD4 is expressed in microglia associated with human glioblastoma samples An analysis of the TCGA profiling data reveals a significant increase in SMAD4 mRNA levels in brain glioblastoma tissue as compared to normal non-malignant tissue **(A)**. Panel shows expression of SMAD4 in human glioblastoma tumor tissue (GB 34) **(B)**. Note that there is evident expression of SMAD4 (red) expression in Iba1 positive microglial cells (green). Cell nuclei are labelled with DAPI (blue). Scale=30μm (*low magnification*), Scale=10μm (*high magnification*). Table depicts the percentage of Iba1 positive microglial cells per tumor **(C)**.

In light of the above results, we sought to validate the expression of SMAD4 in human glioblastoma tissue to better understand its role in glioma pathogenesis. An analysis of the expression profiling data of glioblastoma samples in The Cancer Genome Atlas database using Oncomine software showed a significant increase (3.271 fold) in the expression of SMAD4 (Reporter ID: 202527_s_at) mRNA in glioblastoma tissues (n=542) when compared with non-malignant brain tissue samples available in the database (Figure 3A) [39].

In the present study, double immunofluorescence analysis was performed to determine SMAD4 protein expression in glioblastoma tissues (Figure 3B). Confocal imaging showed expression of SMAD4 in Iba1-positive microglia, as well as non-microglial cells in different tissue samples (Figure 3B). The Iba1-immunoreactive cells appeared to be an amoeboid or rounded phenotype, indicative of activated state of the cell type. High magnification images show evident nuclear expression of SMAD4 in Iba1 positive microglia (Figure 3B). Quantitative analysis of Iba1-positive cell bodies revealed a high percentage of microglia in the tumor samples (Figure 3C).

### SMAD4 regulates the expression of tumor supportive factor MMP9

**Figure 4:**
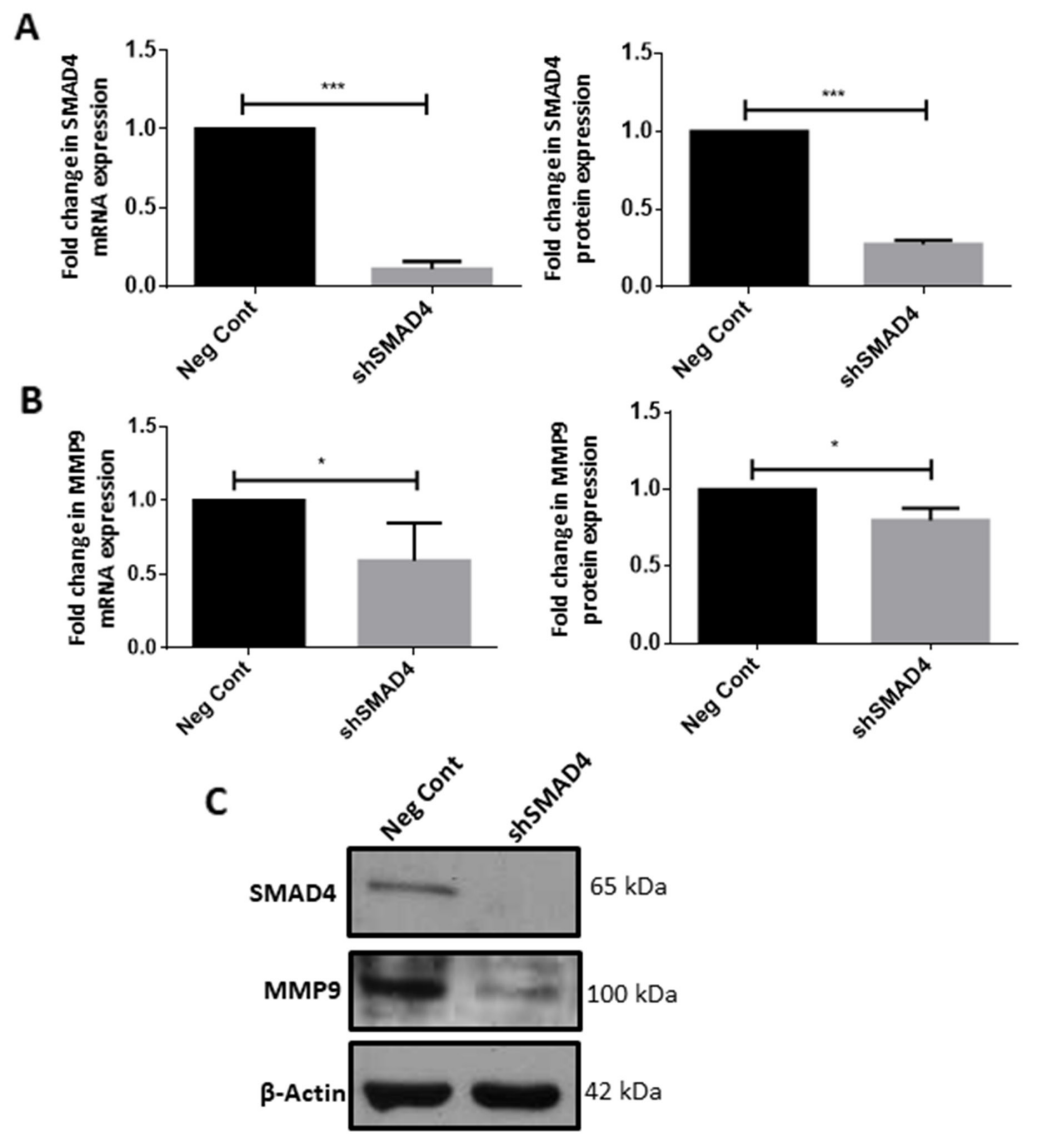
SMAD4 regulates expression of tumor supportive factor MMP9 in microglia Quantitative RT-PCR and western blot analyses shows a decrease in mRNA and protein expression of SMAD4 in shSMAD4 BV2 cells as compared to negative control **(A, C)**. Data represent Mean±SD, (n=3), Students *t*-test, ^***^p<0.001. Quantitative RT-PCR and western blot analyses show that shRNA-mediated knockdown of SMAD4 resulted in a decrease in the mRNA and protein expression of MMP9 **(B, C)**. Mean±SD, (n=4), Students *t*-test, ^*^p<0.05.

In order to ascertain the role of SMAD4 in microglia, stable knockdown of SMAD4 in microglia was carried out by transduction of small-hairpin RNA (shRNA) against the *Smad4* gene. ShRNA-mediated silencing of the *Smad4* gene resulted in 90% decrease in mRNA levels of *Smad4* (Figure 4A) and 80% decrease in the protein level of SMAD4 (Figure 4A, 4C). A downregulation of MMP9 at mRNA and protein level was observed in microglia after knockdown of SMAD4 compared to cells transduced with the empty vector, which served as a negative control (Figure 4B, 4C). This suggests that SMAD4 regulates the expression of tumor supportive factor, MMP9 in microglia.

### ShRNA-mediated silencing of SMAD4 suppresses the migration of microglia towards glioma conditioned medium

**Figure 5:**
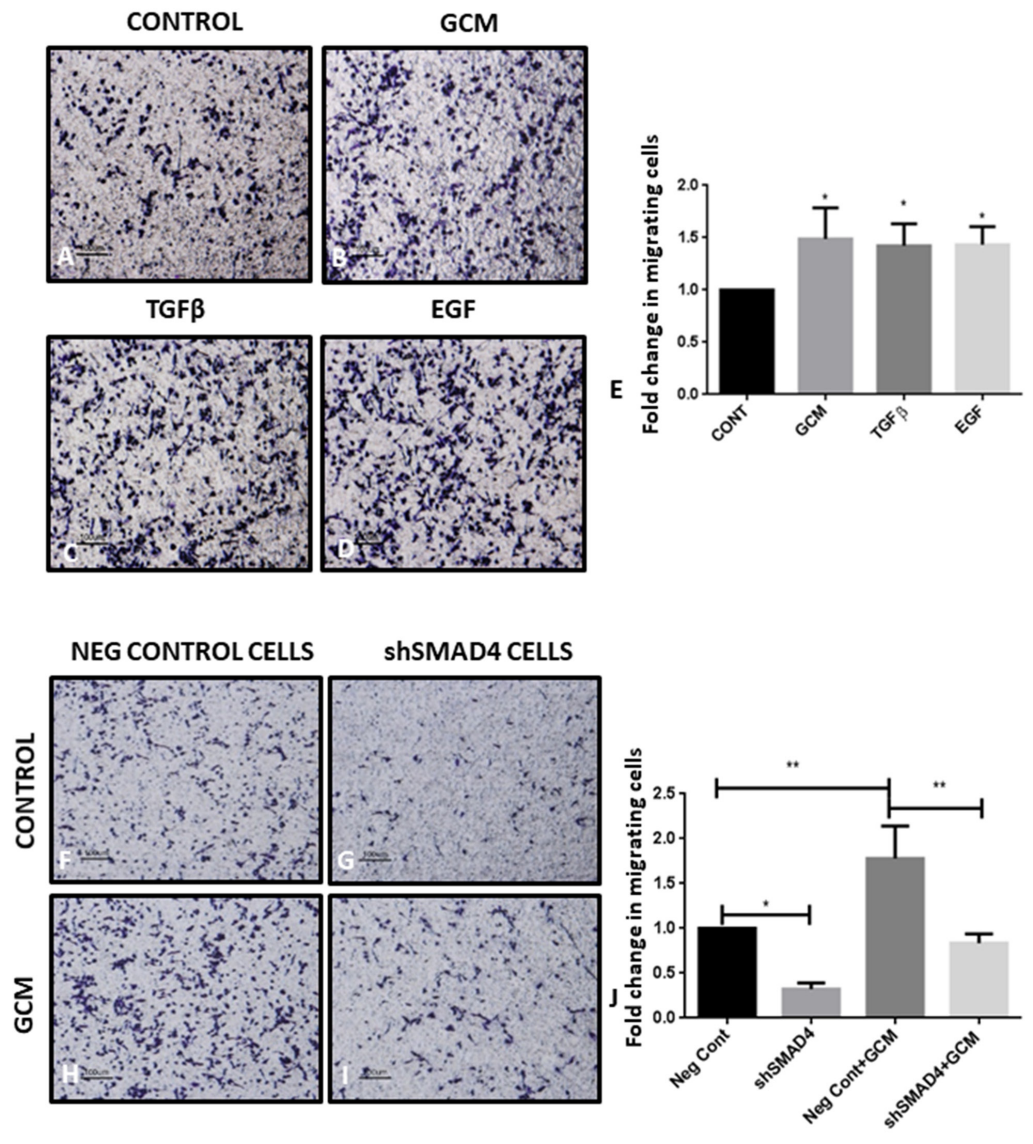
shRNA-mediated knockdown of SMAD4 suppresses microglial migration Trans-well membrane image panels show an increase in the number of migrated microglial cells (*purple*) after exposure to GCM **(B)**, TGFβ **(C)** and EGF **(D)** as compared to cells exposed to serum containing medium **(A)**. Histogram depicts fold change in migrating cells exposed to GCM, TGFβ and EGF as compared with control group **(E)**. Data represent mean±SD, ^*^p<0.05, ^**^p<0.01, (n=3). There was a decrease in the number of migrated shSMAD4 microglial cells (*purple*) in response to GCM and serum containing medium **(I, G)** as compared to negative control (Neg Cont) microglial cells exposed to GCM and serum containing medium **(H, F)**. Histogram depicts fold change in migrating cells **(J)**. Data represent mean±SD, (n=3), ^*^p<0.05, ^**^p<0.01.

The transwell migration assay was performed, wherein microglial cells were seeded in serum-free medium in the upper chamber of a transwell insert and allowed to migrate towards the lower chamber containing GCM or chemoattractants such as TGFβ and EGF, to assess the migratory potential of microglia towards GCM (Figure 5A-5D). There is a significant increase in the number of microglial cells migrating towards GCM (Figure 5B, 5E) in the lower chamber as compared to the number of microglial cells migrating to serum containing medium which served as a control (Figure 5A). In addition, microglial cells showed increased migration towards chambers containing soluble TGFβ and EGF (Figure 5C, 5D, 5E) as compared to control.

Further, the role of SMAD4 in the migration of microglia towards GCM was studied. A significant decrease in the number of shSMAD4 cells migrating towards the serum containing medium in the lower chamber, was observed as compared to control cells which were transfected with empty vector (Figure 5F, 5G, 5J). This indicates that SMAD4 plays a significant role in the migratory potential of microglia. On the other hand, upon exposure to GCM, there was an increase in the number of migrating control and shSMAD4 microglial cells compared to that of control and shSMAD4 microglial cells, respectively. However, the increase in migration of shSMAD4 microglial cells exposed to GCM was significantly less than that of control microglial cells exposed to GCM (Figure 5H, 5I, 5J). In addition, GCM-induced migration index of both control and shSMAD4 microglial cells appear to be comparable (Figure 5J), suggesting that factors involving TGFβ pathway present in GCM could play a role in migration of microglia.

### Microglial conditioned medium from shSMAD4 cells inhibits glioma viability

**Figure 6:**
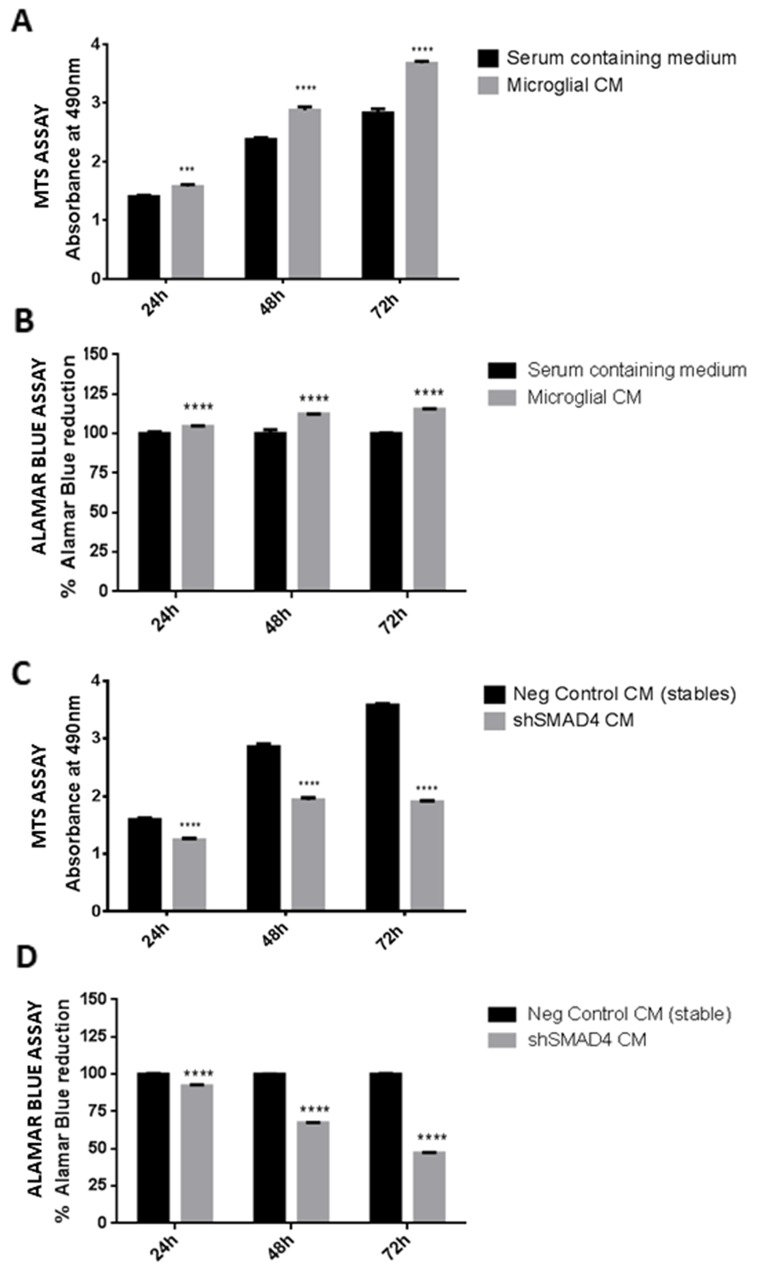
Effect of conditioned medium derived from shSMAD4 knockdown microglial cells on glioma cell viability Histogram depicts MTS assay absorbance values at 24, 48 and 72h after treatment of glioma cells with conditioned medium derived from microglia. A significant increase in glioma viability was observed after treatment with microglia conditioned medium at all time points **(A)**. This was further confirmed using alamar blue viability assay wherein an increase in % of reduction of alamar blue was observed in glioma cells treated with microglia conditioned medium at all time points **(B)**. Histogram further shows the MTS assay absorbance values at 24, 48 and 72h treatment of glioma cells with conditioned medium derived from shSMAD4 microglial cells **(C)**. A decrease in absorbance values indicates a decrease in viability of glioma cells after treatment with conditioned medium from shSMAD4 cells as compared to glioma cells treated with negative control medium (C). In addition, alamar blue assay results **(D)** show a decrease in percentage of alamar blue reduction in glioma cells treated with medium from shSMAD4 microglia as compared to glioma cells treated with negative control medium. Data represent Mean±SD, (n=4), ^***^p<0.001, ^****^p<0.0001.

Microglial cells have been shown to promote glioma progression through secretion of factors that aid in tumor growth. In the present study, the viability of glioma cells in response to conditioned medium from microglia was evaluated using an MTS assay and alamar blue assay. The results indicate that there is a significant increase in cell viability (Figure 6A, 6B) of glioma cells after 24, 48 and 72h of treatment with medium derived from control microglial cells, confirming that microglial cells secrete factors that promote glioma cell growth. In order to determine the effect of knockdown of SMAD4 in microglia on glioma cell viability, glioma cells were treated with conditioned medium derived from shSMAD4 microglial cells. The results indicate that there is a decrease in viability of glioma cells when treated with medium from SMAD4 knockdown microglial cells (Figure 6C), suggesting that suppression of SMAD4 in microglia decreases the viability of glioma cells. This was further confirmed with an alamar blue assay wherein a significant decrease in reduction of alamar blue was observed *in vitro* after treatment of glioma cells with medium derived from shSMAD4 microglial cells as compared to the corresponding control (Figure 6D).

### MiR-146a regulates SMAD4 expression in GCM treated microglia

**Figure 7:**
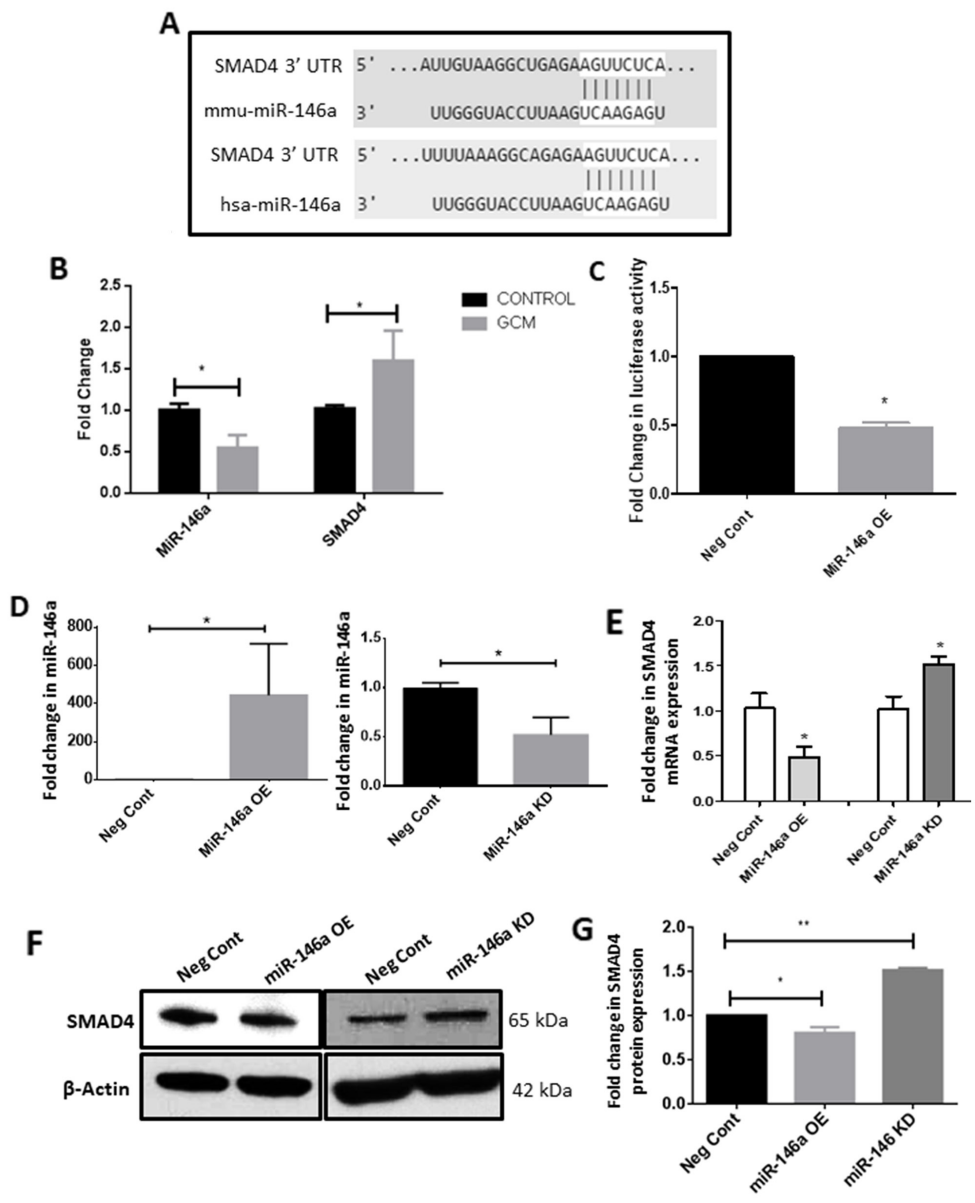
MiR-146a is downregulated in GCM treated microglia and targets SMAD4 TargetScan software predicted that miR-146a putatively binds to 3’UTR of SMAD4 mRNA **(A)**. Histogram depicts a significant decrease in the levels of miR-146a and a significant increase in mRNA levels of SMAD4, indicating an inverse relationship between SMAD4 and miR-146a in microglia treated with GCM **(B)**. Histogram shows a significant decrease in the luciferase activity in cells co-transfected with miR-146a mimic and luciferase vector as compared to cells co-transfected with scrambled control miRNA and luciferase vector, indicating that miR-146a targets SMAD4 **(C)**. Data represent mean±SD, (n=3), Students *t*-test, ^*^p<0.05. MiRNA mimics and inhibitor transfection efficiency was verified using qRT-PCR. Histogram shows an about 400-fold increase in miR-146a levels in microglia after mimic transfection and about 50% decrease in miR-146a levels after inhibitor transfection **(D)**. Data represent mean±SD, (n=3), Students *t*-test, ^*^p<0.05. Histogram shows a decrease in the mRNA levels of SMAD4 upon overexpression of miR-146a and conversely an increase in SMAD4 mRNA upon inhibition of miR-146a **(E)**. Data represent mean±SD, (n=3), Students *t*-test, ^*^p<0.05. Western blot **(F)** and densitometry analysis **(G)** confirm that overexpression of miR-146a suppresses SMAD4 protein levels and inhibition of miR-146a increases SMAD4 protein level as compared to scrambled probe transfected cells. Data represent mean±SD, (n=3), Students *t*-test, ^*^p<0.05, ^**^p<0.01. MiR-146a OE- MiR-146a overexpression; MiR-146a KD-MiR-146a knockdown.

In order to delineate the epigenetic mechanisms that regulate SMAD4 expression in glioma-associated microglia, we examined the 3’UTR of the *Smad4* mRNA sequence to identify miRNA binding sites. TargetScan algorithms software (http://www.targetscan.org/mmu_71/) revealed putative complimentary binding site for miR-146a in the 3’UTR of *Smad4* (Figure 7A). Given the role of miR-146a in activation of microglia [36, 40, 41], its role in the tumor supportive phenotype of microglia was determined. The quantitative RT-PCR analysis showed that the expression level of miR-146a-5p was significantly decreased in glioma-associated microglia as compared to control cells (Figure 7B). This is concurrent with an increase in mRNA and protein levels of SMAD4 (Figure 7B, 2A), demonstrating an inverse relationship between miR-146a and SMAD4 in microglia.

Further, 3’UTR luciferase assay was performed to confirm that SMAD4 is a target of miR-146a. BV2 microglial cells were transfected with a luciferase vector containing the 3’UTR of the mouse *Smad4* gene together with miR-146a overexpression (mimics) or scrambled probes. A significant decrease in the luciferase activity in BV2 microglia was observed upon co-transfection of the mimics and the luciferase vector, indicating that the miR-146a binds to the 3’UTR of *Smad4* in luciferase vector (Figure 7C). Transfection efficiency of the miRNA overexpression and inhibition was determined by examining the levels of the miR-146a in microglial cells after transfection of mimics and inhibitors. Overexpression of miR-146a mimics was observed to increase miR-146a levels in microglia by nearly 400-fold while inhibition of the miR-146a resulted in a ~50% decrease in miR-146a levels (Figure 7D). The mRNA expression of SMAD4 after overexpression and inhibition of the miR-146a was analyzed. MiR-146a mimic transfection resulted in decrease in mRNA and protein expression levels of SMAD4 and conversely, inhibition of miRNA function resulted in an increase in the mRNA and protein levels of SMAD4 (Figure 7E, 7F, 7G) in microglia, suggesting that miR-146a directly targets SMAD4 in microglia.

**Figure 8:**
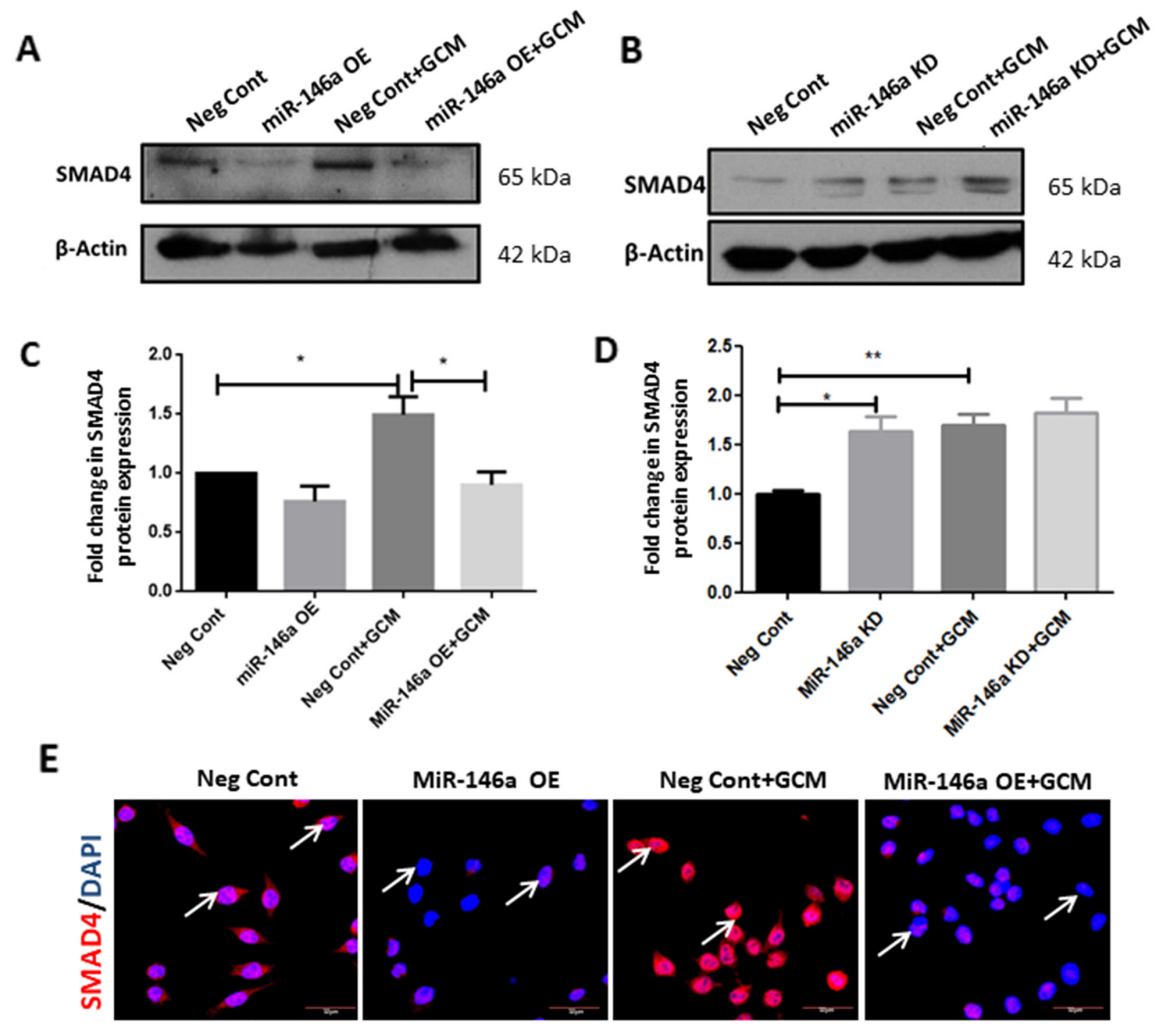
MiR-146a regulates SMAD4 in GCM-treated microglia Western blot analyses show that transfection of microglial cells with miR-146a mimics suppressed the GCM induced induction of SMAD4 protein **(A, C)** Data represent mean±SD, (n=3), ^*^p<0.05. MiR-146a inhibition increases the protein levels of SMAD4 upon GCM treatment of microglia as seen by western blot analyses and densitometry quantification **(B, D)**. Data represent mean±SD, (n=5), ^*^p<0.05. Immunofluorescence labelling shows that miR-146a overexpression attenuated SMAD4 expression (red) in BV2 microglia nuclei with or without GCM treatment as compared to cells transfected with scrambled miRNA (Neg Cont) probes **(E)**. DAPI-nuclei staining, blue. Scale bar=30μm. MiR-146a OE- MiR-146a overexpression; MiR-146a KD-MiR-146a knockdown.

In order to understand the functional relationship between miR-146a and SMAD4 in GCM-induced microglia, loss-of and gain-of function experiments were carried out. MiR-146a overexpression was found to significantly decrease SMAD4 expression at protein levels in microglia treated with GCM, as determined by western blot (Figure 8A, 8C). In contrast, inhibition of the miR-146a resulted in a marginal increase in the protein levels of SMAD4 in microglia treated with GCM as revealed by western blot analyses (Figure 8B, 8D). Immunocytochemistry analysis also revealed that miR-146a overexpression with mimics decreased the expression of SMAD4 in microglia treated with or without GCM as compared to that transfected with scrambled probes (Figure 8E). This suggests that miR-146a regulates SMAD4 expression in glioma-associated microglia.

### MiR-146a regulates the expression of MMP9 in microglia and suppresses migration of microglia towards GCM

**Figure 9:**
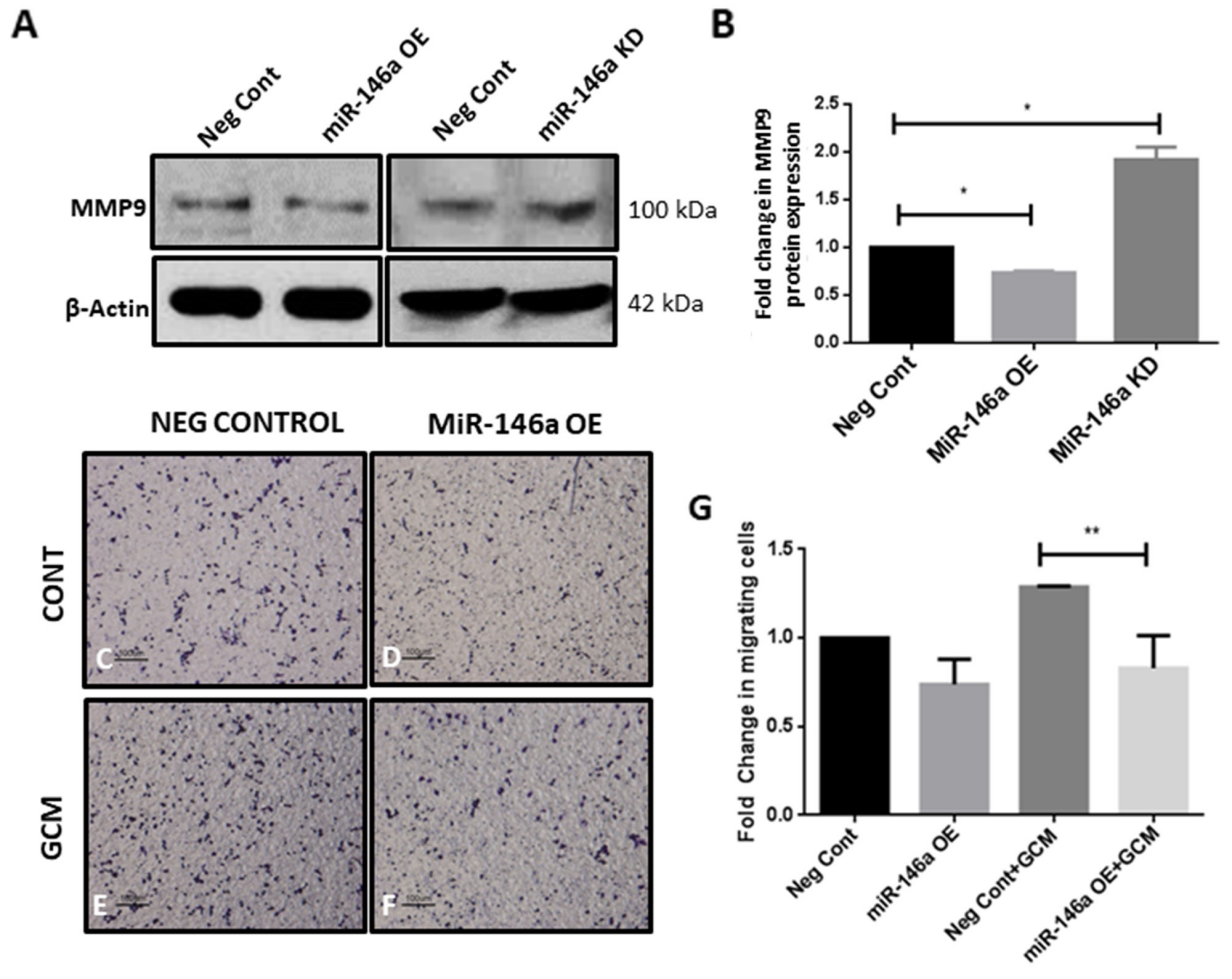
MiR-146a alters expression of tumor supportive gene MMP9 in glioma associated microglia and suppressed microglial migration towards GCM Overexpression of miR-146a suppressed expression of MMP9 at the protein level and conversely, inhibition of miR-146a in microglia was found to increase levels of MMP9 as depicted by immunoblotting and densitometry analysis **(A, B)**. Data represent mean±SD, (n=3), Students *t*-test, ^*^p<0.05. Image panel shows a decrease in the number of migrated microglial cells (*purple*) after overexpression of miR-146a in response to GCM as compared to Neg Cont cells **(C-F)**. Histogram depicts fold change in migrating cells **(G)**. Data represent mean±SD, (n=3), ^**^p<0.01. MiR-146a OE- MiR-146a overexpression; MiR-146a KD-MiR-146a knockdown.

Microglia treated with GCM showed an induction of tumor promoter gene MMP9, concomitant with an increase in SMAD4 level. In order to determine if miR-146a regulates tumor supportive gene expression in microglia, expression of MMP9 upon overexpression and knockdown of miR-146a was analyzed. Overexpression of the miR-146a in microglia resulted in a significant suppression of MMP9 protein expression while inhibition of miR-146a was found to increase MMP9 expression (Figure 9A, 9B). In addition, overexpression of miR-146a in microglia resulted in a significant decrease in migration of microglia towards GCM in a transwell migration assay (Figure 9C-9G), indicating that miR-146a suppresses microglial migration through regulation of SMAD4 and its downstream gene MMP9.

### miR-146a overexpression in microglia suppresses glioma viability and growth

**Figure 10:**
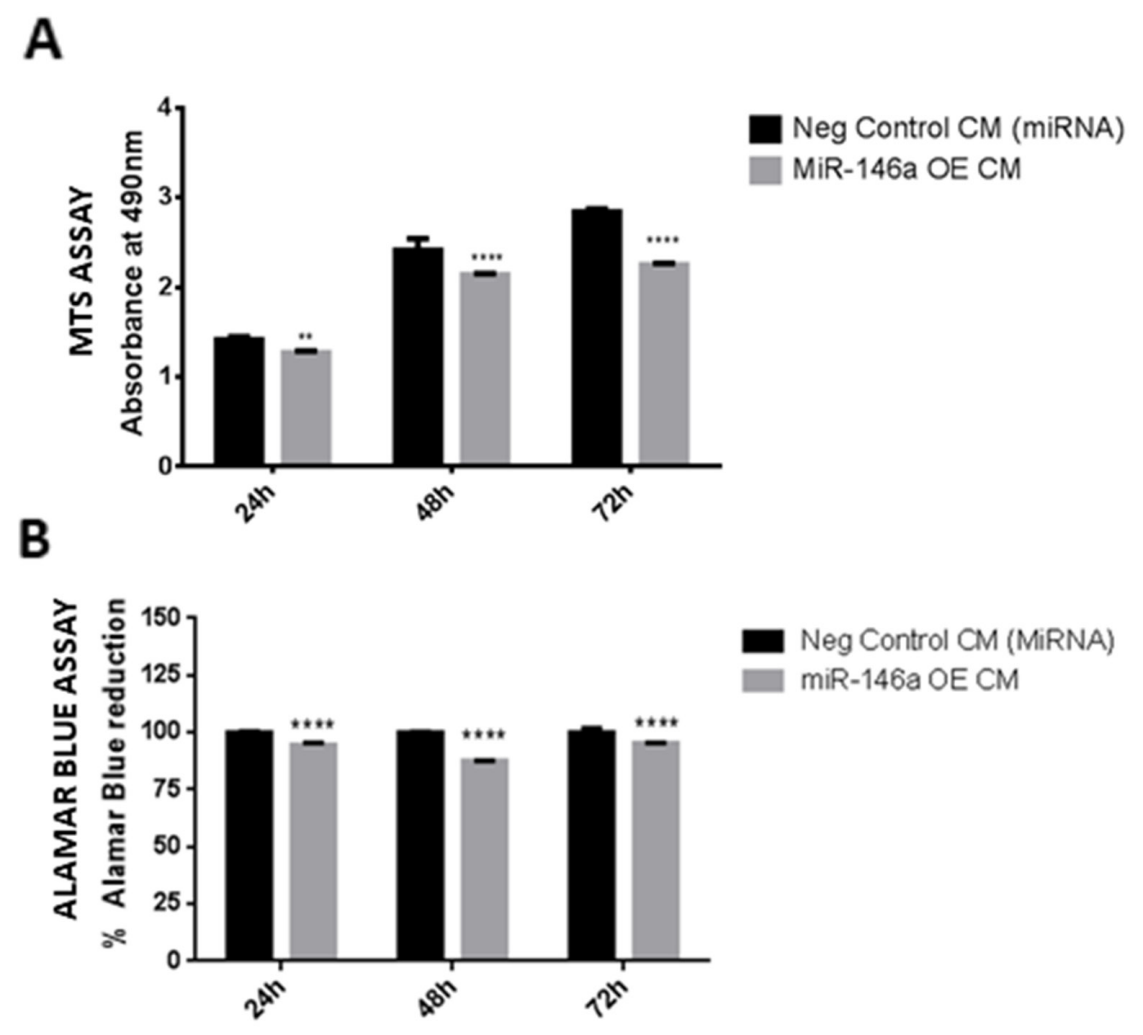
Effect of conditioned medium derived from miR-146a overexpression in microglia on viability of glioma cells Histogram depicts MTS assay absorbance values 24, 48 and 72h treatment of glioma cells with conditioned medium derived from miR-146a overexpression in microglia. A decrease in absorbance values at all time points indicates an inhibition of viability of glioma cells after treatment with medium from miR-146a overexpressed cells as compared to glioma cells treated with negative control (scrambled miRNA) transfected medium **(A)**. Further, alamar blue assay results show a decrease in percentage of alamar blue reduction, indicating a suppression of glioma cell viability upon treatment with medium from miR-146a overexpressed microglia as compared to glioma cells treated with scrambled control transfected medium **(B)**. Data represent Mean±SD, (n=4), ^**^p<0.01, ^****^p<0.0001.

To determine the effect of miR-146a overexpression in microglia on glioma cell viability, glioma cells were treated with conditioned medium derived from miR-146a overexpressed microglial cells. The MTS results indicate that there is a decreased viability of glioma cells across different time points after treatment with miR-146a overexpressed microglia-derived medium as compared with that of scrambled control transfected cells (Figure 10A). This was further confirmed with an alamar blue assay wherein a significant decrease in alamar blue reduction was observed *in vitro* after treatment of glioma cells with medium from miR-146a overexpressed microglial cells (Figure 10B), indicating that overexpression of miR-146a in microglia decreased the viability of glioma cells.

## DISCUSSION

It is well documented that microglia play diverse roles, either detrimental or beneficial, during CNS pathology [6]. In the normal healthy brain, microglia monitor the brain microenvironment for pathogens and injury and are involved in functions such as neuronal synapse formation, maintenance and pruning [42–44]. Upon activation, the microglia with processes rapidly transform into amoeboid phenotype and are involved in phagocytosis of debris in brain parenchyma [45]. In the present study, microglia within gliomas exhibit primarily amoeboid phenotype, suggesting that they are activated, probably in response to the glioma secretome which consists of signaling molecules released by neoplastic and non-neoplastic cells such as vascular endothelial cells, astrocytes and cancer stem cells of the tumor that lie in spatial proximity to microglial cells. This is supported by recent experimental evidence, which showed that microglia exposed to glioma conditioned medium *in vitro* and microglia associated with glioma tumors in mice models *in vivo* exhibit an amoeboid phenotype that is characteristic of a state of activation [46–48]. Further, the glioma tumors analyzed in the present study showed a high percentage of Iba1-positive microglial cells, with certain glioblastoma tumors hosting nearly 25%-50% of microglial cells in the tumor mass. A higher frequency of microglial cells in the tumor may be attributed to migration of microglia in the brain parenchyma in response to factors released by the glioma cells as can be seen in the *in vitro* migration assay. Several studies have shown that soluble factors such as EGF [49, 50] and TGFβ [51] serve as potent chemotactic factors in tumors and may promote migration of microglia towards the tumor as observed in the present study.

In addition to its role as a chemoattractant, the TGFβ ligand activates an anti-inflammatory signaling pathway in microglia, exerting an opposing effect on pro-inflammatory signaling that is widely known to be neurotoxic to brain tissue [52, 53]. TGFβ has been shown to act on microglia in an autocrine manner and maintain microglial quiescence [54]. It has also been shown that microglia-derived TGFβ enhanced the invasiveness and tumorigenicity of the glioma cells and siRNA-mediated knockdown of TGFβ Receptor II in glioma cells disrupted this tumor promoting effect of TGFβ [30]. The present study shows increased levels of total and pSMAD2/3, which mediate TGFβ signaling pathway, in microglia after GCM treatment *in vitro*. While the roles of SMAD2/3 have been widely studied in microglia [55, 56], the role of SMAD4 in microglial activation, specifically in context of the TGFβ signaling has remained unclear. There is evidence showing that SMAD4 is upregulated in LPS-activated microglia and acts as a negative feedback inhibitor of NFκB, a pro-inflammatory signaling response in the activated microglia [57]. In the present study, glioma-associated microglia expressed SMAD4 in human glioblastoma tumors *in vivo* and microglia exposed to GCM showed increased expression of SMAD4 *in vitro*. This was further supported by an analysis of the data in The Cancer Genome Atlas which revealed an upregulated SMAD4 level in glioblastoma tumors as compared to normal brain samples. Further, shRNA-mediated silencing of SMAD4 in microglia was associated with a decrease in the expression of MMP9, an extracellular matrix metalloproteinase and suppression of microglial migration. This has been evidenced in hepatocellular carcinoma wherein knockdown of SMAD4 reduced migratory capacity and colony formation ability of the cancer cells [26]. SMAD4 has also been shown to control endodermal cell migration during embryonic development through regulation of extracellular matrix modelling enzymes, MMP9 and MMP14 [58]. In addition, SMAD4 silencing in pancreatic tumor cells and keratinocytes has been shown to abolish TGFβ induced migration [59], therefore highlighting a vital role for SMAD4 in microglial migration.

Microglial cells are highly secretory in nature. In the present study, microglia-derived growth factors in the conditioned medium were found to promote glioma cell viability *in vitro*. This is an important finding as it may lead to a novel therapeutic strategy which may focus on suppressing microglia-mediated tumorigenesis. Moreover, this study demonstrates that glioma-associated microglia promote tumorigenesis through SMAD4 expression, since glioma cells treated with conditioned medium derived from shSMAD4 microglial cells showed decreased viability. This decreased viability of glioma cells is also possible *via* SMAD4-induced negative regulation of NF-κB pathway in microglia, since recent studies have shown that SMAD4 knockdown in microglia induced pro-inflammatory cytokine, IL-6 in an NF-κB dependent manner [57]. Overall, these results indicate that the possible interaction between SMAD4 and NF-κB in glioma-associated microglia may determine the tumor progression.

The epigenetic regulation of SMAD4 was investigated, as these mechanisms provide an additional layer of post-transcriptional control. In this study, SMAD4 was found to be targeted by miR-146a in microglia. MiR-146a, a miRNA enriched in immune cells, is known to be upregulated in activated microglia and macrophages in pathological conditions such as infection [41], ischemic stroke [60] and Alzheimer's disease [40, 61]. Several studies have shown that miR-146a modulates the innate immune response of activated microglial cells through regulation of the pro-inflammatory transcription factor, NFκB [41, 62]. Recently, the miRNA-146b, which shows sequence similarity with miR-146a has been shown to inhibit glioma growth *in vitro* through modulation of its target EGFR [63]. Downregulation of miR-146a in glioma-associated microglia as observed in the present study may favour tumorigenesis through increased expression of SMAD4 and its downstream genes which are involved in tumor progression. This also indicates that the glioma-associated microglia do not exhibit activated microglial phenotype as observed in other neuropathologies such as Alzheimer's and stroke. Functional studies further confirmed that miR-146a is a negative regulator of tumorigenic gene expression in microglia *via* its target SMAD4, as its overexpression in microglia resulted in suppression of MMP9, which is a tumorigenic factor that promotes migration of microglia towards GCM and the glioma viability *in vitro*.

**Figure 11:**
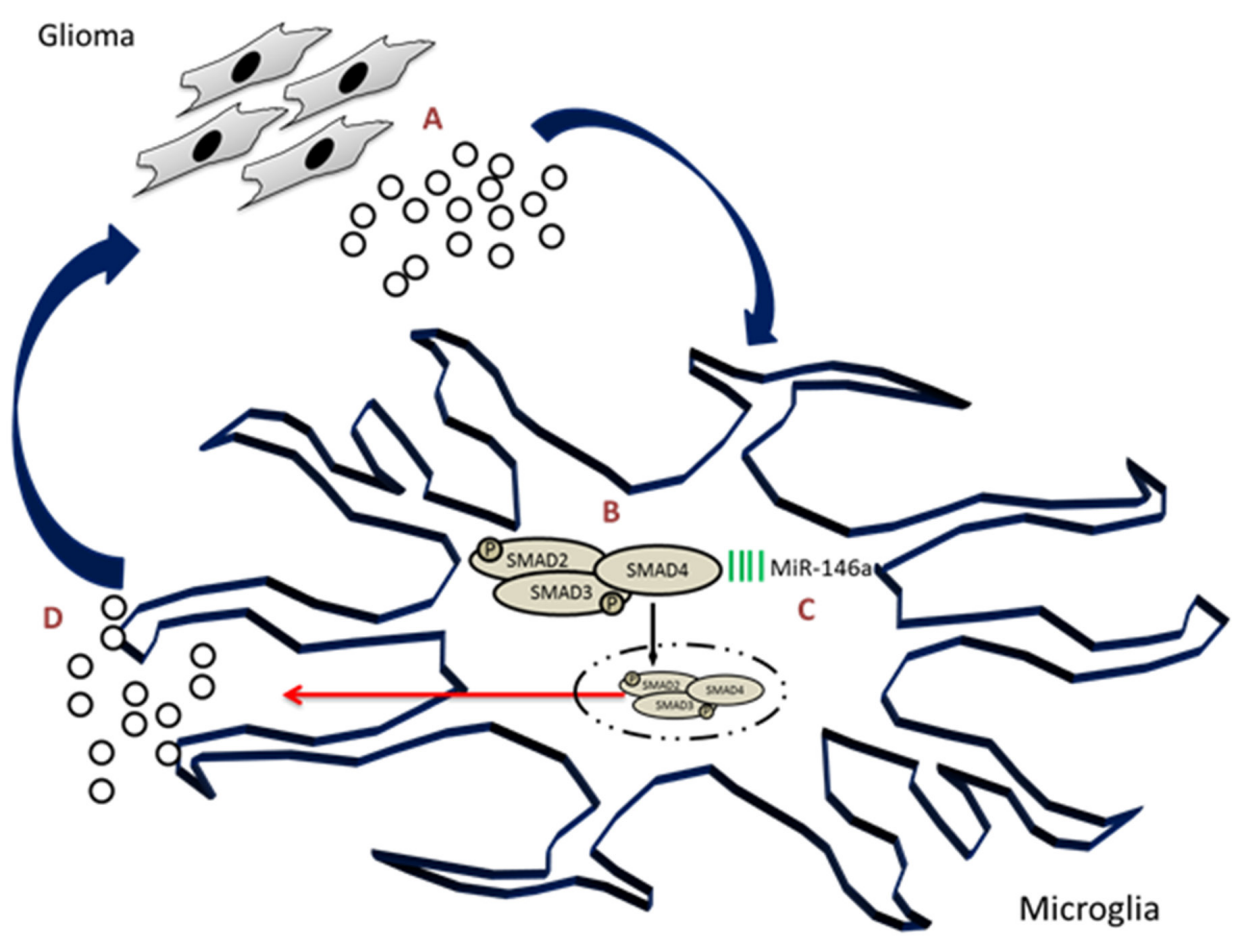
SMAD4, regulated by microRNA-146a, promotes microglial cell migration and tumor progression in glioma environment Glioma cells secrete a number of factors including TGFβ **(A)** that activates the TGFβ pathway in microglia associated with glioma. Glioma conditioned medium was found to induce phosphorylation of SMAD2/3 complex and increase the level of SMAD4 **(B)** in microglia. Concurrently, miR-146a which was found to target SMAD4, was downregulated in glioma associated microglia **(C)**. Downregulation of miR-146a in glioma-associated microglia increases the levels of tumor supportive factors **(D)**, including SMAD4 and MMP9 which promote glioma progression and microglial migration.

As the immune cell of the brain, microglia show complex phenotypes in the brain tumor and did not reject or phagocytose the tumor cells. Instead, they conglomerate within the tumor core and support the tumor progression. It is thus imperative to study microglial function in context of different molecular and genetic subtypes of glioblastoma. In this regard, this study shows robust expression of SMAD4 in glioblastoma tumors and in glioma-associated microglia. It has also been demonstrated that SMAD4 which was found to be post-transcriptionally regulated by miR-146a, regulates the migration of microglial cells in response to glioma conditioned medium. This study further established that miR-146a suppresses tumorigenic gene, MMP9 in glioma-associated microglia and glioma cell viability through its target SMAD4 (Figure 11). Further *in vivo* studies are required to evaluate the therapeutic effect of miR-146a and SMAD4 on glioma growth and progression.

## MATERIALS AND METHODS

### Human tissue samples

Graded brain tumor specimens were obtained with written informed consent, as part of a study protocol approved by the SingHealth Centralised Institutional Review Board A and the National Healthcare Group Domain-Specific Review Board A (Table 1). All protocols were approved by the Institutional Review Board, National University of Singapore. Use of glioblastoma human tissues was reviewed and approved by the National University of Singapore Institutional Review Board (NUS-IRB Reference Code: B-16-049E).

**Table 1:** Tumor sample grade and type

Sample Name	Tumor Type	Tumor Grade
GB-21	Glioblastoma	grade IV
GB-22	Glioblastoma	grade IV
GBO-24	Glioblastoma with oligodendroglial component	grade IV
GB-25	Glioblastoma	grade IV
GBO-26	Glioblastoma with oligodendroglioma	grade IV
GB-28	Glioblastoma	grade IV
GB-34	Glioblastoma	grade IV

### Immunofluorescence

Human tumor sections were fixed, frozen, sectioned (7μm thick) and mounted onto slides. Tissue sections were blocked in 3% bovine serum albumin (BSA) solution in phosphate-buffered-saline (PBS) containing 0.3% Triton-X (TX). Primary antibody against the full length human SMAD4 protein was a kind gift from Dr. Lu Lei from the Nanyang Technological University, Singapore. Sections were incubated with primary antibodies in 1% BSA overnight at 4°C at the following concentrations: SMAD4-1:300, Iba1-1:50 (Abcam, ab5076). Further, sections were stained with 4’,6-diamidino-2-phenylindole (DAPI) (1:5000) and mounted with a fluorescent mounting medium. Fluorescence images were captured using a confocal microscope (Olympus, FV1000 Fluoview). Iba1 positive cell bodies showing a well-defined DAPI stained nuclei were manually counted across 4 fields of the tumor section and plotted as a percentage of total DAPI cell nuclei.

### Cell culture

BV2 murine microglial cells and C6 rat astrocytoma cells obtained from American Tissue type Culture Collection (ATCC) were cultured in DMEM supplemented with 10% fetal bovine serum (FBS). Cells were maintained in a 5% CO_2_ incubator at 37°C and regularly passaged with Trypsin-EDTA solution to allow healthy growth of cells.

### Preparation of glioma conditioned medium (GCM)

To mimic a glioma microenvironment *in vitro*, microglial BV2 cells were treated with conditioned medium from the C6 cell line [64, 65]. Briefly, C6 cells were seeded in 10cm culture dishes at a density of 2×10^6^ cells. Cells were allowed to settle overnight and DMEM supplemented with 10% FBS was added to the culture the next day. Culture supernatant was collected after 48h and filtered through a 0.22μm filter to remove cellular debris. GCM was stored at -80°C and freeze-thaw cycles were minimized.

### Generation of SMAD4 knockdown stable cells in microglia

Stable knockdown of SMAD4 was performed by lentiviral mediated transduction of SMAD4 specific shRNA in BV2 microglial cells. Microglial cells were transduced with 4 shRNA individual clones (Dharmacon, GE Healthcare) against the SMAD4 gene (Accession Number: NM_008540). Selection pressure was applied 48h after transduction using puromycin at the concentration of 2μg/ml. Cells were maintained in puromycin containing medium for 6-10 days and expanded [66]. Efficiency of knockdown in microglia was confirmed using western blotting analysis and the shSMAD4 clone that induced maximal knockdown, shSMAD4_2, was selected for further analysis (Supplementary Figure 1).

### RNA extraction, cDNA conversion and quantitative real-time PCR (miRNA)

RNA was isolated from the BV2 cells after transfection or treatment, using miRNeasy Mini Kit (Qiagen, Cat No: 217004) as per the manufacturer's instructions. RNA isolate (10ng) was used for conversion of miRNA to cDNA using Universal cDNA Synthesis Kit II (Exiqon, Cat No: 203301). Quantification of miRNA expression in control and GCM treated groups was carried out using ExiLENT SYBR^®^ Green master mix (Exiqon, Cat No: 203421). LNA-modified miR-146a-5p primers (Exiqon, Cat No: 204688) and small nuclear U6 RNA primers (Exiqon, Cat No: 203907) were used for miRNA real time PCR.

### cDNA conversion and quantitative Real-time PCR (mRNA)

The total RNA (2000ng) was converted to cDNA for gene expression analysis using a master mix consisting of M-MLV Reverse transcriptase enzyme (Promega, Cat No: M170A), M-MLV Reverse Transcriptase 5X Reaction Buffer (Promega, Cat No: M531A), dNTP (Promega, Cat No: U1511) and RNasin^®^ Ribonuclease inhibitor (Promega, Cat No: N2111) in a 25μl reaction volume. The qRT-PCR for gene expression was performed using Fast SYBR Green Master Mix (ThermoFisher Scientific, Cat No: 4385614) with 1:10 ratio dilution of cDNA in nuclease-free water. Primer sequences for PCR reactions are given in Table 2. Fold change between control and experimental groups was calculated as per 2^-ΔΔCt^ method [67]. All PCR reactions were carried out in a real time Applied Biosystems PCR system (Life Technologies, Model No: 7900HT).

**Table 2:** Primer sequences

Gene	Forward Primer	Reverse Primer
SMAD4	TCCAACACCCGCCAAGTAAT	GCTGGCTGAGCAGTAAATCC
MMP9	GCGTGTCTGGAGATTCGACTT	TATCCACGCGAATGACGCT
β-Actin	GGATTCCATACCCAAGAAGGA	GGATTCCATACCCAAGAAGGA

### Migration assay

The Transwell migration assay was carried out to assess the migratory potential of microglia towards GCM, TGFβ and EGF. 40,000 microglial cells were seeded in the upper chamber of the Transwell migration insert (Corning, Cat No:3422) and was placed in the lower chamber containing glioma conditioned medium and medium containing TGFβ (PeproTech, Cat No 100-21C) and human recombinant EGF (Life Technologies, PHG0311). The cells were allowed to migrate for 15h. Inserts were fixed in 100% methanol for 10min and stained for visualization using 0.5% cresyl violet solution. Cells on the upper membrane of the insert were removed using a cotton swab. 5 random fields from 3 biological replicates were quantified and the results are plotted as fold change between treatment and control groups.

### Transfection of miRNA mimics and inhibitors

BV2 cells were plated at a density of 2×10^5^ in 6-well plates and gain-of and loss-of-function studies of miR-146a-5p was carried out using miRNA mimics (Ambion, Cat No: 4464066) and miRNA inhibitors (Exiqon, Cat No: 4100679-001). BV2 cells were also transfected with scrambled mimics (Ambion, Cat No: 4464058) or inhibitors (Exiqon, Cat No: 199006-001) as negative controls. The mimic and inhibitors complexes for transfection were prepared using X-tremeGENE siRNA Transfection Reagent (Roche, Cat No: 04476093001) in Opti-MEM medium at concentrations of 20 and 40nM, respectively. RNA and protein analyses were performed at 48h and 72h post transfection, respectively.

### Western blotting

Protein extracts from BV2 microglial cells were obtained using a cocktail of M-PER^™^ Mammalian Protein Extraction Reagent (ThermoFisher Scientific, Cat No: 78501), Halt^™^ Protease Inhibitor (ThermoFisher Scientific, Cat No: 78430) and phosphatase inhibitor (ThermoFisher Scientific, Cat No: 78427). Protein quantification was performed by Bradford assay using the Bio-Rad protein Assay Kit (Bio-Rad, Cat No: 5000001). Total protein lysate (30μg) was denatured at 95°C for 10min. Proteins were loaded onto a 10% SDS-polyacrylamide gel (PAGE) electrophoresis setup and transferred to membranes (Bio-Rad, Cat No: 162-0177). Blocking of non-specific sites on the membrane was done using 5% milk or bovine serum albumin (BSA). Membranes were incubated overnight at 4°C with primary antibodies as follows: SMAD4-1:1000 (Cell Signaling Technology, Cat No: 9515), VEGFa-1:500 (SantaCruz, Cat No: sc-152) and MMP9-1:2000 (EMD Millipore, Cat No: AB19016) and subsequently incubated with horseradish peroxidase conjugated secondary antibodies (ThermoFisher Scientific, Cat No: 31430, Cat No: 31460) 1h. Pico Chemiluminescent substrate (ThermoFisher Scientific, Cat No: 37070) was used to develop the blots and the protein expression level was quantified densitometrically (Bio-Rad Quantity One^®^ 1-D Analysis Software, Cat No: 1709600).

### Luciferase assay

A luciferase assay was performed to verify if miR-146a targets SMAD4 mRNA. BV2 microglial cells were plated at a density of 20,000 cells in 24 well plates. The luciferase vector containing the 3`UTR of SMAD4 was commercially purchased from GeneCopoeia (Cat No: MmiT027594). Cells were co-transfected with mimics and scrambled probes (30nm) and luciferase vector (1000ng) and the medium containing secreted luciferase was collected at 24h. The Secrete-Pair Dual Luminescence Assay Kit (GeneCopoeia Cat No: SPDA-D010) was used to determine luminescence of secreted *Gaussia* luciferase (GLuc). A secondary reporter, secreted alkaline phosphatase (SEAP), served as an internal control. GLuc/SEAP ratio was determined to measure the luminescence output of the transfected sample.

### Immunocytochemistry

BV2 microglial cells were grown on poly-L-lysine coated coverslips. Following transfection and/or treatment, cells were fixed with 4% paraformaldehyde for 15min at room temperature. Permeabilization of cell membranes was achieved using 0.1% Triton-X containing PBS. Following this, the slides were blocked using 5% normal goat serum and incubated overnight at 4°C with the following antibodies- SMAD4-1:100 (Santa Cruz, sc-7966), pSMAD2/3-1:200 (Cell signaling technology, Cat No: 8828) and SMAD2/3-1:200 (Cell Signaling Technology, Cat No: 8685). Following this, cells were incubated with fluorophore tagged secondary antibodies: anti-rabbit Cy3 (Sigma, Cat No: c2306), anti-mouse Cy3 (Sigma, Cat No: C2181) and FITC conjugated lectin (Sigma, Cat No: L0401) was used as a marker for microglial cells. Cell nuclei were counterstained with DAPI for visualization. Fluorescence images were captured using a confocal microscope (Olympus, FV1000 Fluoview).

### ELISA based quantification of TGFβ in GCM

TGFβ in the C6 glioma conditioned medium (GCM) was quantified using the Quantikine ELISA Kit (RND Systems, MB100B) as per the manufacturer's instructions. Briefly, latent TGFβ1 was activated to its immunoreactive form by addition of 20μl of 1M HCl to 100μl of the GCM, and subsequently neutralized by adding 20μl of 1.2N NaOH. The TGFβ1 standard was reconstituted and serially diluted using the diluent solution provided in the kit. 50μl of GCM and the TGFβ1 standard samples were added to the TGF-β1 antibody pre-coated ELISA plate and incubated for 2h. Subsequently, the samples were discarded, and the wells were thoroughly washed using wash buffer. Next, 100μl of the TGFβ1 conjugate was added to each well and incubated for 2h. Following washing, the plate was incubated with substrate solution, and reaction was stopped using the stop solution provided in the kit. The optical density was measured at 450nm using a plate reader.

### MTS and alamar blue cell viability assay

In order to evaluate the effect of SMAD4 knockdown in microglia on glioma cell viability, an MTS and alamar blue assay was performed. Conditioned medium was collected from control microglia (microglial CM) and from microglia after knockdown of SMAD4 (shSMAD4-CM) or miR-146a overexpression (MiR-146a OE-CM). Conditioned medium from microglia transduced with the empty vector (Neg Control CM (stables)) and the scrambled probes (Neg Control CM (miRNA)) served as controls. About 10,000 of C6 glioma cells were seeded in 96-well plates and treated with the conditioned medium from different groups. Post 24, 48 and 72h of treatment, 20μl of MTS reagent (Promega, Cat No. G358C) was added to the cells and incubated at 37°C for 2h. Following this, absorbance was read at 490nm in a 96-well plate reader. The alamar blue assay was performed by adding 10μl of the alamar blue reagent (ThermoFisher Scientific, Cat No. DAL1100) to the cells after treatment with conditioned medium. Absorbance was read at 570 nm and normalized against absorbance at 600nm. The results are plotted as a percentage of alamar blue reduction.

### Statistical analysis

Data from at least three biological replicates were analyzed using GraphPad Prism and Microsoft Excel software and represented as mean ± S.D. In comparing 2 experimental groups, the Student's *t*-test was used. Multiple groups were analyzed using one-way or two-way ANOVA tests, followed by *post-hoc* Tukey's and Sidak's test. Data sets were considered significant at *p*<0.05.

## SUPPLEMENTARY MATERIALS AND FIGURES


