# Supplementary material for: Microglial SMAD4 regulated by microRNA-146a promotes migration of microglia which support tumor progression in a glioma environment
Source: Oncotarget. 2018 May 18;9(38):24950–69. doi: 10.18632/oncotarget.25116 (PMC5982777; doi:10.18632/oncotarget.25116)
Supplement: Supplementary file 1 [file oncotarget-09-24950-s001.pdf]

Microglial SMAD4 regulated by microRNA-146a promotes migration of microglia which support tumor progression in a glioma environment

SUPPLEMENTARY MATERIALS

A

	CLONE ID
shSMAD4_1	TRCN0000025881
shSMAD4_2	TRCN0000025885
shSMAD4_3	TRCN0000025900
shSMAD4_4	TRCN0000025953

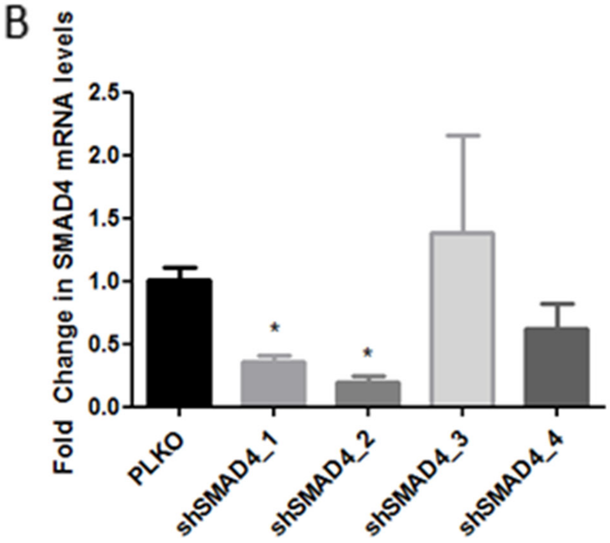

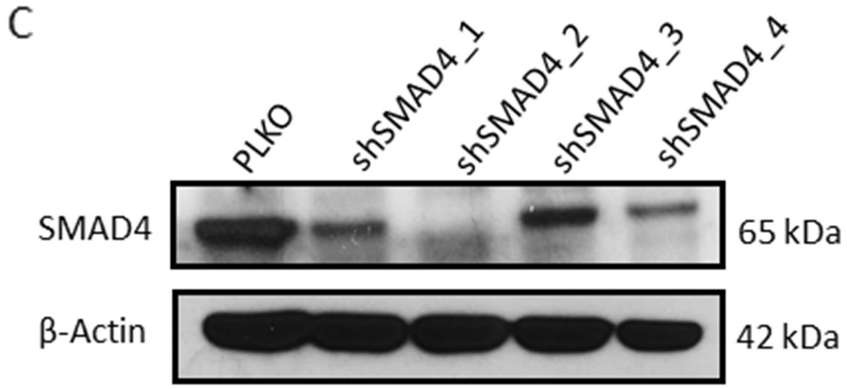

Supplementary Figure 1: Table shows the shRNA clones that were used for stable knockdown of SMAD4 in microglia **(A)**. Quantitative RT-PCR and Western Blot analysis depicts the expression of SMAD4 after transduction of 4 clones of shSMAD4 in microglia **(B, C)**.
